# Evaluating the Confidence of Non-specialty Doctors Working in Trauma and Orthopedics in Performing Knee Arthrocentesis Through Simulation-Based Teaching

**DOI:** 10.7759/cureus.71314

**Published:** 2024-10-12

**Authors:** Swechhya Banstola, Neil Ashwood, Adam T Stammer, Veylamuthen Murugan, Adrian Crawford

**Affiliations:** 1 Trauma and Orthopedics, Queen's Hospital Burton, University Hospitals of Derby and Burton NHS Foundation Trust, Burton-on-Trent, GBR; 2 Research Institute, University of Wolverhampton, Wolverhampton, GBR; 3 Trauma and Orthopedics, University Hospitals of Derby and Burton NHS Foundation Trust, Burton-on-Trent, GBR

**Keywords:** arthrocentesis, knee joint, medical education, orthopedics, simulation

## Abstract

Introduction

Acute monoarthropathies that present in emergency settings include septic arthritis, where urgent joint arthrocentesis is the diagnostic gold standard. Literature indicates low confidence among trainee doctors in performing knee aspirations. Simulation-based teaching can be used to supplement procedural skills training and improve their confidence in performing such procedures.

Methods

This study aimed to assess the self-rated confidence of non-specialty doctors (N=8) in Trauma and Orthopedics in conducting knee aspirations using simulation-based teaching with an anatomically accurate knee model. Pre- and post-intervention questionnaires investigated self-reported confidence using a 10-point Likert-type scale and participant experience using a 7-point Likert scale. Pre- and post-intervention surveys further qualitatively explored attitudes toward conducting the skill.

Results

Pre-intervention mean confidence was rated 3.9 (SD=2.70) out of 10, with a noted increase to 8.1 (SD=1.25) out of 10, providing a mean difference of 4.2 (SD=2.82) out of 10 with p=0.007. All attendees agreed or strongly agreed on the usefulness and satisfaction of the activity. Qualitative analysis indicated themes of nervousness and lack of confidence pre-intervention and attitudes of increased confidence in the skill post-intervention.

Conclusions

Overall, increased statistically significant confidence was concluded among non-specialty doctors in conducting knee arthrocentesis following simulation-based teaching, with perceived usefulness and satisfaction of the activity. Nonetheless, we must consider potential limitations of clinical accuracy and realism through such procedural skills training.

## Introduction

Acute monoarthropathies are commonly encountered presentations within hospital admissions teams. Defined as the inflammation of one joint, predominantly the knee, the presentation is often characterized by joint swelling, warmth, and pain and can be accompanied by pyrexia [1]. Causes of acute monoarthropathies include gout and septic arthritis, with both increasing in reported incidence of 63.44% worldwide from 1999 to 2019 and 43% in the United Kingdom (UK) from 1998 to 2013, respectively [2-4]. Delayed management of septic arthritis holds significant consequences of osteomyelitis, irreversible joint destruction, and death, with a 90-day mortality rate of 7.05%, increasing to 22.69% in those aged over 79 when admitted as a primary diagnosis [5].

Urgent arthrocentesis for synovial fluid analysis remains the gold standard investigation for septic arthritis, emphasizing its role in differentiating from gout, and it holds a prominent role in targeted antibiotic therapy [2,6]. Patients presenting with acute monoarthropathies are often initially managed by trainee doctors; however, a quantitative survey of 140 trainee doctors across two UK hospitals concluded that 52% of participants reported low confidence in managing hot, swollen joints and 27% reported that they did not feel competent at performing the procedure. With 31% reporting inadequate training, we can consider the need for educational interventions of joint aspirations among trainee doctors [7]. The need for adequate training is further reinforced by trainee doctors associating emotions such as ‘weary’ and ‘anxious’ with joint aspirations within a focus group [8].

The training of procedural skills within medical education often parallels Kolb’s theory of experiential learning [9]. In the context of joint aspirations, the aim of such training can enable trainees to reflect on concrete evidence with existing knowledge of the skill, undergo reflective observation as they observe knee aspirations, form abstract conceptualization to develop a better understanding of conducting the procedure, and lastly, engage in active experimentation when applying this skill practically. This further coincides with common evidence-based teaching of procedural skills following the six steps of learn, see, practice, prove, do, and maintain [10].

Although trainees can see and observe procedures within the clinical setting, literature widely supports the role of simulation-based teaching for the practice and development of procedural skills [10]. This provides trainees with low-risk environments, immediate feedback, and repeated application to develop confidence in the desired skill [11]. Concordantly, prior educational interventions using simulation-based teaching proved enhanced confidence in conducting joint aspirations amongst trainees, with one intervention indicating doubled confidence following the learning activity [12,13].

A preliminary questionnaire concluded that most rotating foundation doctors had not completed a knee aspiration during a four-month rotation at our secondary care setting and none had received any structured teaching on the procedure, rating low levels of confidence in conducting the skill (Appendix A).

## Materials and methods

Aim

This study aimed to assess the effect of a simulation-based learning intervention on teaching knee aspiration, with the objective of evaluating self-rated pre- and post-intervention confidence in the skill as well as trainee experience. 

The null hypothesis was that in non-specialty doctors, simulation-based teaching on knee arthrocentesis would result in no difference in self-reported confidence to conduct the skill.

Context and setting

This study was undertaken at the Trauma and Orthopedics Department of Queen’s Hospital Burton, a secondary care setting providing 24-hour acute orthopedics care. Non-specialty doctors within the department partake in the first-on-call position of the acute orthopedics team, often encountering acute knee monoarthropathies. A preliminary questionnaire, using Google Forms (Google LLC, Mountain View, United States), of six non-specialty doctors following a rotation in Trauma and Orthopedics concluded no prior teaching of knee arthrocentesis and low confidence in conducting the procedure, self-rated 2.5 (SD=1.64) and 1.67 (SD=0.82) out of 10 in conducting and teaching the skill, respectively (Appendix A).

With the inevitable future encounters of knee monoarthropathies during specialist training in general practice and medical or surgical pathways, the importance of developing a learning intervention to improve the confidence of non-specialty doctors to conduct knee arthrocentesis prevailed.

Developing the learning intervention

A one-hour learning workshop was developed, focusing on delivering teaching about knee arthrocentesis. This was delivered by a Core Trainee 1 (CT1) in Trauma and Orthopedics and involved discussion on the presentation, investigations, and management of an acute, hot, swollen joint, followed by demonstration and guidance on conducting the knee aspiration. This involved an explanation for preparing a sterile field and equipment tray. Next, a demonstration of how to identify surface landmarks for deducing aspiration points on the knee was delivered. Then, the educator explained the sites and angles of entry to the synovium, followed by a demonstration of aspiration of the fluid with an aseptic, non-touch technique, and lastly, how samples are handled, including filling sterile sample pots and labeling the samples for laboratory investigations. The learners then practiced the procedure. The model used was a commercially available Limbs and Things model, anatomically accurate for identifying surface landmarks as well as for allowing multiple punctures without marking on the skin layer and with the ability for aspiration of synovial fluid [14]. 

**Figure 1:**
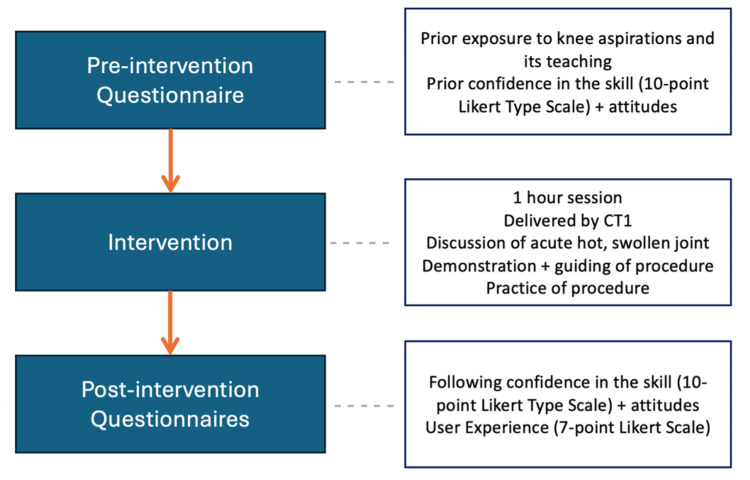
Study methods CT1: Core Trainee 1 doctor

Figure 1 demonstrates the structure of the learning intervention. Learning was supplemented by incorporating a developed guide by the authors for this project (Appendix B) [15-18].

Assessment of learning

A mixed-methods study approach was undertaken with a combination of outcomes exploring qualitative and quantitative outcome measures.

Outcomes and outcome measures

Outcomes and outcome measures were based on the Kirkpatrick model of training evaluation, notably used to evaluate learning interventions, representing Levels 1 to 4, with reaction, learning gain, behavior, and results, respectively [19].

Level 1 investigating reaction explored the experience of the learners following the intervention. This was rated on a post-intervention questionnaire using a 7-point Likert scale for the phrase ‘I enjoyed this learning activity’, where 1 = strongly disagree, 2 = disagree, 3 = somewhat disagree, 4 = neutral, 5 = somewhat agree, 6 = agree, and 7 = strongly agree. Other phrases were also evaluated including ‘The activity was useful for my learning’, ‘The activity was delivered well’, and ‘Overall, I am satisfied with the activity’ using the same scoring scale.

Open instructions ‘Please use the space below to share your thoughts on how you feel about conducting knee aspirations before this session’ in the pre-intervention questionnaire and ‘Please use the space below to share how you feel about conducting a knee aspiration following the session’ in the post-intervention questionnaire were also asked to consider pre- and post-intervention attitudes toward conducting knee aspirations.

Level 2 investigating learning gain was evaluated by comparing learners’ self-reported confidence in the skill at baseline and following the intervention using pre- and post-intervention questionnaires (Appendix C, Appendix D). Learners were asked to rate their confidence from 1 to 10 (1 = least confident, 10 = most confident) for the questions ‘How would you rate your confidence in conducting a knee aspiration?’ and ‘How would you rate your confidence in teaching someone how to conduct a joint aspiration?’ prior to and following the session.

Level 3 and Level 4 behavior and results, respectively, were not investigated, with the primary focus of the study being on improving learner confidence in conducting the skill.

The primary outcome of the study was the evaluation of self-rated confidence in conducting knee aspirations (pre and post). Secondary outcomes for the study were differences in self-rated confidence in conducting knee aspirations, self-rated confidence in teaching knee aspirations (pre and post), attitudes toward conducting knee aspirations (pre and post), and trainee experience of the learning intervention (assessing enjoyment, usefulness, delivery, and satisfaction).

Data analysis

Level 1: Reaction - Learner Experience

The data collected to assess students’ scoring of questionnaire phrases were interpreted without further statistical analysis. A thematic analysis was conducted on participants’ responses to open-ended questions.

Level 2: Learning Gain

The study used pre- and post-intervention questionnaires with Likert-type scales evaluating self-rated confidence in conducting and teaching knee aspiration. IBM SPSS Statistics (IBM Corp., Armonk, United States) was used for data analysis. Mean differences and standard deviations of self-rated confidence in conducting and teaching knee aspirations were calculated. We could not assume a specific normal distribution of the ranked scores, hence to assess statistical significance, the non-parametric Mann-Whitney U test was conducted, with calculated p-values less than 0.05 considered as statistically significant.

Ethics

As part of a quality improvement project, to increase the confidence of non-specialty doctors in performing knee arthrocentesis, this study did not require further ethical approval.

## Results

The table of results can be identified in Appendix E.

Participants

Two workshops were conducted, spanning two four-month rotations, consisting of a total of eight non-specialty doctors. Of this, five (62.5%) were Foundation Year 2 (FY2) doctors, two (25%) were Junior Clinical Fellow (JCF) doctors, and one (12.5%) was a CT1 doctor.

Learner baseline

**Figure 2:**
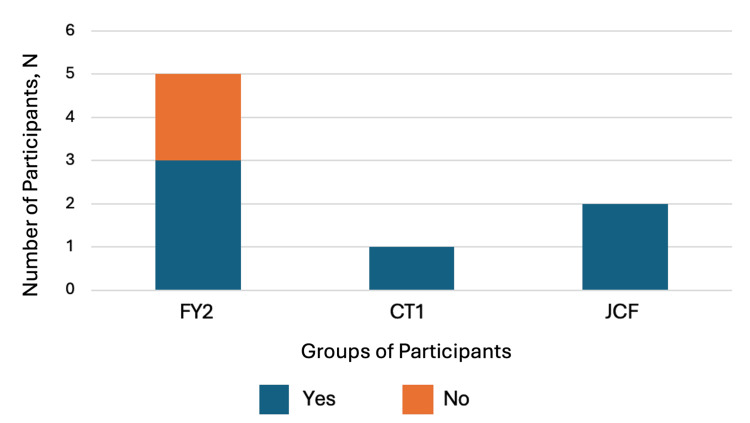
Responses to 'Have you observed any knee aspirations?' in pre-intervention questionnaire, with options 'Yes' or 'No', among groups of participants separated by grade X-axis: Groups of participants - FY2 (N=5), CT1 (N=1), JCF (N=2). Y-axis: Number of participants (N) answering 'Yes' or 'No'. JCF: Junior Clinical Fellow doctors; FY2: Foundation Year 2 doctors; CT1: Core Trainee 1 doctor

The learner baseline was assessed using the pre-intervention questionnaire (Appendix C). As noted in Figure 2, of the eight non-specialty doctors, six (75%) had prior observed knee aspirations, whereas two (25%), both of whom were FY2 doctors, had not. Of the eight total participants, five (62.5%) had prior completed knee aspirations, and three (37.5%) participants, all FY2 doctors, had not. Three (37.5%) of the eight non-specialty doctors had received prior informal teaching on conducting knee aspirations but five (62.5%) had not. No participant had received any formal teaching on conducting knee aspirations previously. 

Learning gain

A 10-point Likert-type scale was used to assess self-rated confidence prior to and following simulation-based teaching using pre- and post-intervention questionnaires, where 1 = least confident and 10 = most confident.

**Figure 3:**
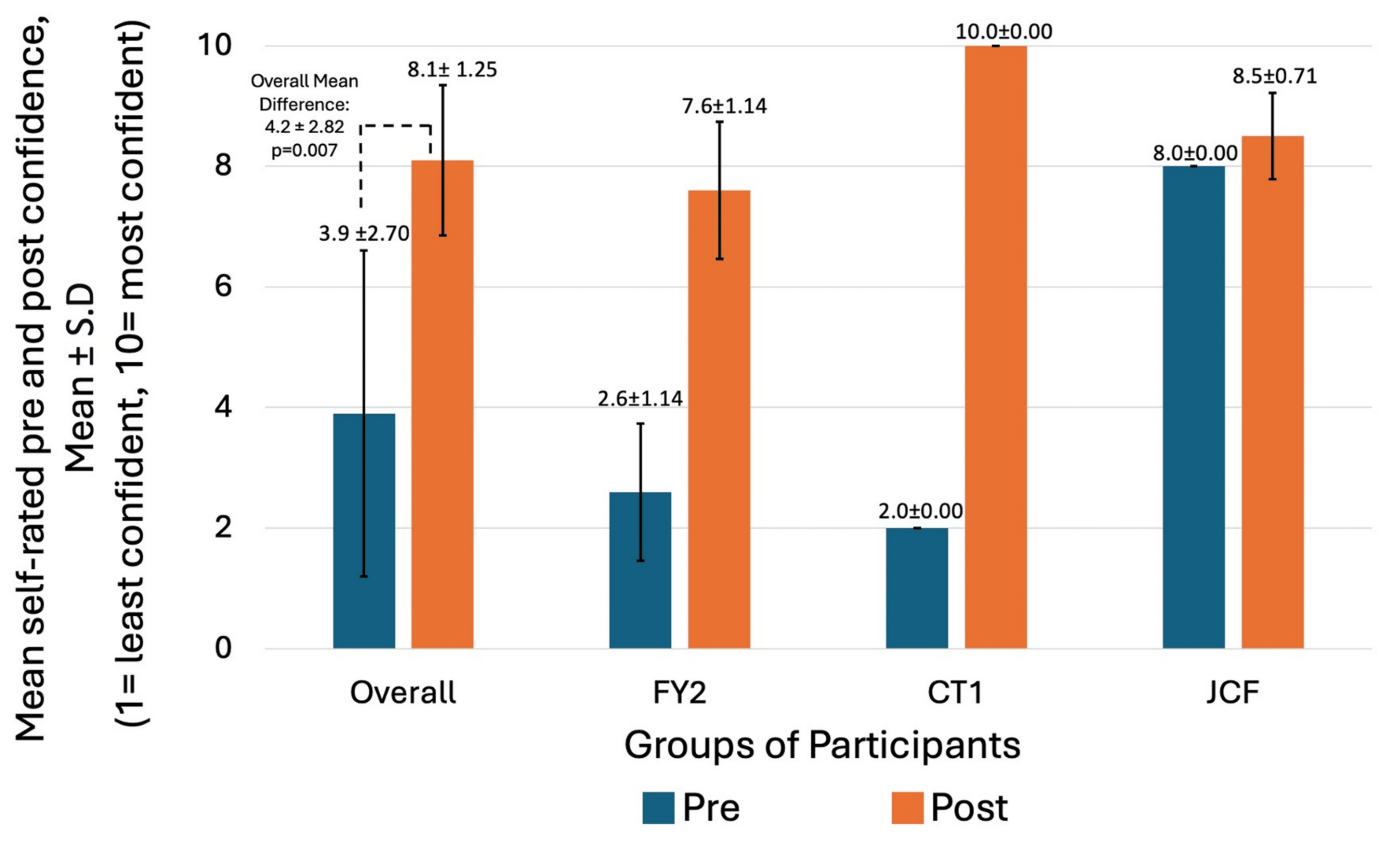
Comparing mean self-rated confidence in conducting knee arthrocentesis pre- and post-intervention (1 = least confident, 10 = most confident) X-axis: Groups of participants - Overall (N=8), FY2 (N=5), CT1 (N=1), JCF (N=2). Y-axis: Participant mean self-rated confidence in conducting knee arthrocentesis at the time of pre- and post-intervention questionnaires evaluated on a 10-point Likert-type scale, where 1 = least confident, 10 = most confident. JCF: Junior Clinical Fellow doctors; FY2: Foundation Year 2 doctors; CT1: Core Trainee 1 doctor

**Figure 4:**
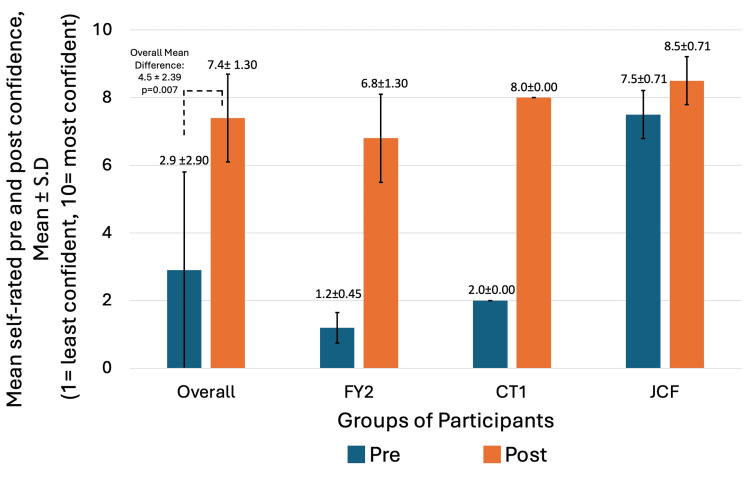
Comparing mean self-rated confidence in teaching knee arthrocentesis pre- and post-intervention (1 = least confident, 10 = most confident) X-axis: Groups of participants - Overall (N=8), FY2 (N=5), CT1 (N=1), JCF (N=2). Y-axis: Participant mean self-rated confidence in teaching knee arthrocentesis pre- and post-intervention questionnaires evaluated on a 10-point Likert-type Scale, where 1 = least confident, 10 = most confident. JCF: Junior Clinical Fellow doctors; FY2: Foundation Year 2 doctors; CT1: Core Trainee 1 doctor

Figure 3 and Figure 4 indicate pre- and post-intervention mean confidence in conducting and teaching knee aspirations, respectively, across FY2, CT1, and JCF, as well as overall. The pre-intervention questionnaire showed an overall mean self-rated confidence in conducting knee aspirations of 3.9 (SD=2.70) out of 10 and an overall mean confidence in teaching knee aspirations of 2.9 (SD=2.90) out of 10. Post-intervention questionnaire showed overall self-rated mean confidence in conducting knee aspiration and teaching knee aspiration of 8.1 (SD=1.25) and 7.4 (SD=1.30), respectively. An overall mean difference in self-rated confidence in conducting and teaching knee aspirations was 4.2 (SD=2.82) out of 10 (p=0.007) and 4.5 (SD=2.39) out of 10 (p=0.007), respectively. A statistically significant, with p<0.05, higher confidence in conducting and teaching knee arthrocentesis was noted with simulation training. 

It is important to note the heterogenicity among non-specialty doctors of different grades, from FY2, CT1, and JCF, with pre- and post-self-rated confidence in conducting and teaching knee arthrocentesis.

Learner attitude to knee arthrocentesis

For the pre-intervention questionnaires, the learners were asked to share their thoughts on how they felt about conducting knee aspirations prior to the workshop. Six (75%) out of eight non-specialty doctors completed a response for this. Of the six responses, five (83.3%) were from FY2 doctors, all of whom reported common themes of feeling nervous, not confident, or uncomfortable. One response (16.7%) of the six, by a JCF doctor, reported feeling comfortable with the skill. Following the teaching of knee aspirations through simulation teaching, learners were asked again to share how they felt about conducting the skill. Seven (87.5%) out of eight participants responded to this, with responses sharing common themes of being more confident and more capable, as well as the procedure seeming straightforward. One of the two JCF doctors responded that the teaching was useful and concise.

Learner experience

Learner experience was explored as part of the post-intervention questionnaire. All participants responded to the four statements, rating their agreement to the statement out of seven, with results in Table 1. All participants (100%) rated the statement ‘I enjoyed the learning activity’ as 7 out of 7, noting a mean score of 7 (SD=0.00). Seven (87.5%) out of eight participants rated the statement ‘This activity was useful for my learning’ as 7 out of 7, with one participant (12.5%) rating it 6 out of 7, noting a mean score of 6.9 (SD=0.35) out of 7. All participants (100%) rated the statement ‘The activity was well delivered’ as 7 out of 7, noting a mean score of 7 (SD=0.00). Seven (87.5%) out of eight participants rated the statement ‘Overall, I am satisfied with the activity’ as 7 out of 7, with one participant (12.5%) rating it 6 out of 7, noting a mean score of 6.9 (SD=0.35) out of 7. Hence, consistent positive trainee experience was noted within this simulation-based teaching. 

**Table 1:** Learner experience of the learning intervention Learning experience evaluated on the post-intervention questionnaire with a rating of statements on a 7-point Likert scale: (1 = strongly disagree, 2 = disagree, 3 = somewhat disagree, 4 = neutral, 5 = somewhat agree, 6 = agree, 7 = strongly agree) JCF: Junior Clinical Fellow doctors; FY2: Foundation Year 2 doctors; CT1: Core Trainee 1 doctor

Post-Intervention Questionnaire Statements	JCF	JCF	FY2	FY2	FY2	FY2	FY2	CT1	Mean ± SD
I enjoyed the learning activity	7	7	7	7	7	7	7	7	7.0 ± 0.00
This activity was useful for my learning	7	7	7	6	7	7	7	7	6.9 ± 0.35
The activity was well delivered	6	7	7	7	7	7	7	7	6.9 ± 0.00
Overall, I am satisfied with the activity	7	7	7	7	7	7	7	7	7.0 ± 0.35

## Discussion

An increasing number of acute monoarthropathies have been presenting acutely in hospital settings in the last decade [2-4]. Knee arthrocentesis holds an invaluable role as the gold standard in the diagnosis of septic arthritis, a significant cause of acute monoarthropathies [2,6], with initial management often undertaken by non-specialty doctors working in Trauma and Orthopedics. Nonetheless, one study indicated that trainee doctors expressed low levels of confidence in managing acute monoarthropathies as well as conducting knee arthrocentesis; 27% did not feel competent in performing the skill and 31% expressed inadequate training [7]. Likewise, at our site, during the preliminary assessment, non-specialty doctors self-rated low levels of confidence in conducting and teaching knee arthrocentesis, at 2.5 (SD=1.64) and 1.67 (SD=0.82) out of 10, respectively. 

Learning intervention through simulation training provides a low-risk environment for trainees to learn and practice clinical skills, developing confidence in conducting the skill [10,11]. This study involved evaluating the confidence of non-specialty doctors working in Trauma and Orthopedics in performing knee arthrocentesis, using an anatomically accurate knee model, assessing learner experience, learner attitude toward the skill, and learning gain.

Learning gain

Our study demonstrated a mean difference in self-rated confidence in conducting and teaching knee aspirations, with scores among the eight non-specialty doctors as 4.2 (SD=2.82) out of 10 (p=0.007) and 4.5 (SD=2.39) out of 10 (p=0.007), respectively, indicating statistical significance. Within such a period, mean self-rated confidence in conducting the skill roughly doubled, from 3.9 to 8.1. This coincides similarly with a prior study, which revealed doubled confidence in conducting the skills amongst learners following simulation training [13], hence suggesting a positive impact on learning gain through the use of simulation training for teaching knee arthrocentesis.

Learner attitude toward knee arthrocentesis

Our study enabled a comparison of learner attitude to knee arthrocentesis prior to and following teaching through simulation. Similarly to a prior study [8], our study supported findings of initial negative emotions to performing knee aspirations among trainee doctors. Following the workshop, we could conclude a trend of feeling more confident in conducting the skill, suggesting enhanced learner attitude to conducting knee aspirations following simulation training. Further, specific learner attitudes to different components of performing a knee aspiration could be explored, such as identifying landmarks or maintaining sterility, to deduce which aspects learners feel more or less confident in, suggesting areas for further training.

Learner experience

Learner experience enables understanding of the acceptability of a learning intervention. Our study indicated positive learner experience with the use of simulation training for teaching knee arthrocentesis to non-specialty doctors in Trauma and Orthopedics.

This study, hence, concluded that simulation training can improve non-specialty doctors’ confidence in conducting knee aspirations, with statistical significance, further enhancing positive attitudes to knee aspirations following the workshop, with a positive learner experience. Similarly to learner attitude, furthering from our study, learner experience could be explored more through evaluation of trainee experience with different stages of performing a knee aspiration, such as identifying landmarks and maintaining sterility, through the use of such simulation-based teaching. 

Implications

Concluding an increase in learning gain, enhanced attitude, and positive trainee experience, we can suggest that simulation-based teaching using an anatomically accurate knee model is a feasible method to provide knee arthrocentesis teaching to non-specialty doctors in its clinical applicability.

With consideration of implications for future research to further assess the impact of simulation training on teaching knee arthrocentesis, we must consider the previously discussed Kirkpatrick model of training evaluation [19]. The study holds the strength of evaluating both learner attitude/experience and learner gain; however, future implications for research include assessment of changes to behavior among non-specialty doctors, such as translating learning gain to clinical application of the skill. Further, we must also consider measures of the clinical impact of such training, i.e., translation of learning gain and behavioral changes to clinical results, achieving parameters such as time from presentation to diagnosis of septic arthritis. This longer-term evaluation can enable appraisal of the training in its clinical efficacy.

Limitations

Although noting the encouraging impact of such training, with statistically significant improved confidence, we must be mindful of the limitations of our study. Firstly, the sample size of eight, which although accurately represents our studied cohort of non-specialty doctors within our department, may limit the reproducibility and generalisability of results. Therefore, there are limitations to the applicability of our findings to further cohorts and centers. Further, heterogeneity between varying grades of non-specialty doctors in our study resulted in varied prior exposure and baseline confidence in knee arthrocentesis. Therefore, this raises concerns about variability in results if the study is reproduced elsewhere with a differing composition of non-specialty doctors compared to our site, causing a limitation to the generalisability of our results. Considering this, differences in confidence at pre- and post-intervention were illustrated overall as well as specifically among FY2, JCF, and CT1 groups in Figure 3 and Figure 4. With heterogeneity in pre-intervention confidence, such confidence was rated the highest among the JCF doctors than less experienced FY2 doctors, and concurrently, indicated the smallest degree of difference in self-rated confidence among JCF doctors, affecting overall mean differences. Nonetheless, while acknowledging the heterogeneity and varying size of effect, the direction of effect remained consistent, suggesting a positive impact of the training irrespective of the level of the non-specialty doctor.

In view of the heterogeneity within the training, it may have been beneficial to consider more inexperienced participants for the greatest evaluation of the training method, such as to consider the intervention without prior existing experience of the skill. However, this may lead to a greater degree of the effect being noted, deviating from the true effect within our represented cohort of all non-specialty doctors within the studied department. Likewise, as no participant had prior formal/structured teaching of the skill, regardless of the level of the non-specialty doctor, all participants provided invaluable insight into learner experience in the evaluation of the training method. 

Within the study, a further limitation stands with potential response bias. The nature of the study with a pre- and post-intervention structure provides limitations caused by response-shift bias. To reduce this, questionnaires were anonymized. Nonetheless, trainees may anticipate and hence record a positive impact of the learning intervention in such pre- and post-intervention questionnaires. Within our study, this limitation was further exacerbated with trainees and trainers working in the same department, an unavoidable aspect of the study, which may have been reduced through external trainers delivering the intervention. However, this holds the limitation of relying on further trainer enthusiasm.

Considering the clinical application, we must note the limitation of simulation training also relying on user acceptability. To reduce this, a commercially available anatomically accurate knee model was used for the training. Nonetheless, during our study, with open space feedback noting two (25%) of eight participants suggesting they found the model difficult to use and advised using a better model, ongoing concern about realism persisted. This could suggest a potential area for further development of knee aspiration models with a greater degree of realism to support simulation-based teaching for performing knee arthrocentesis.

## Conclusions

Literature provides the rationale for the use of simulation-based teaching for developing learning confidence in clinical skills. Our study notes that simulation-based teaching may provide an effective method for improving the confidence of non-specialty doctors working in Trauma and Orthopedics in performing knee arthrocentesis, with positive attitudes toward performing the procedure and improved learner experience. However, the assessment of behavioral changes following such training and its clinical impact warrant further research.

## Figures and Tables

**Figure 5:**
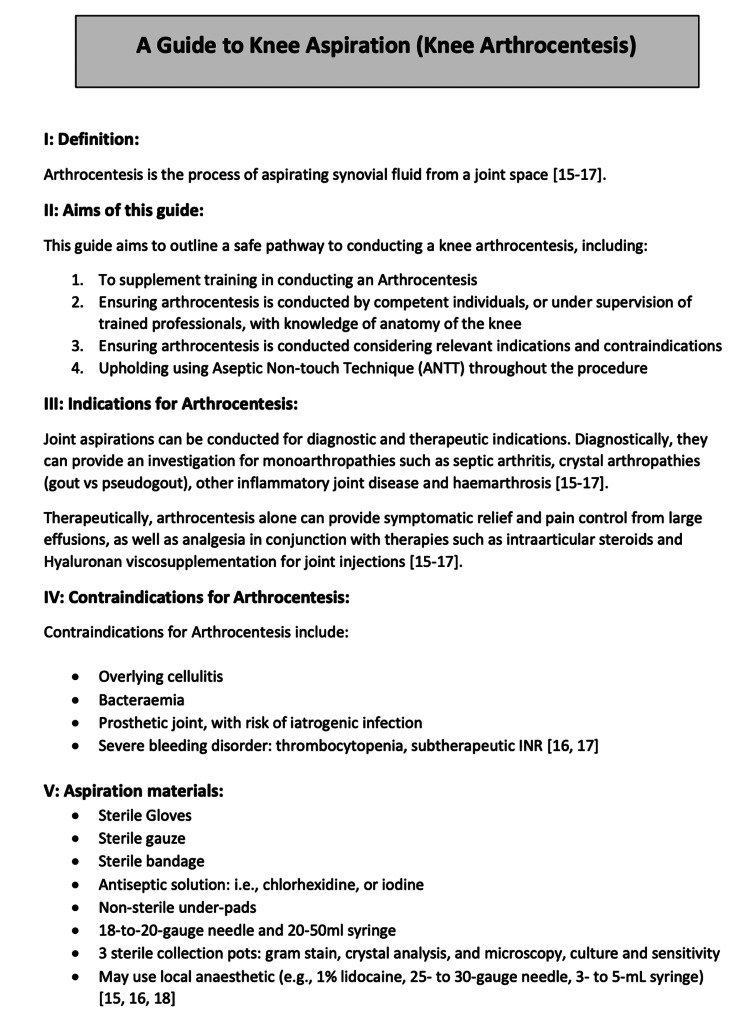
A guide to knee arthrocentesis (1/4)

**Figure 6:**
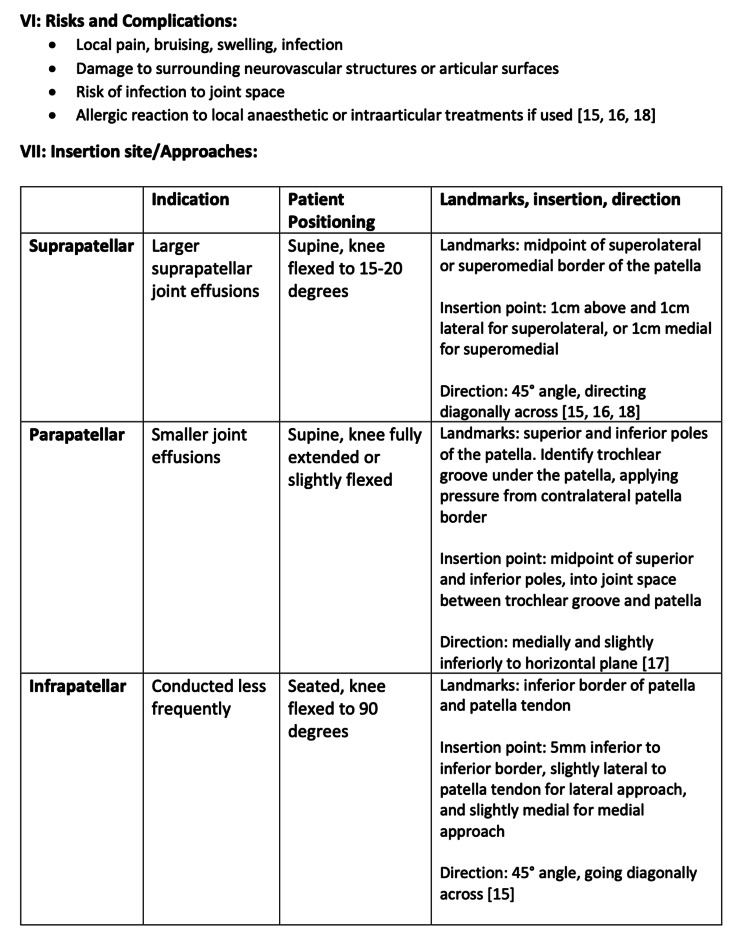
A guide to knee arthrocentesis (2/4)

**Figure 7:**
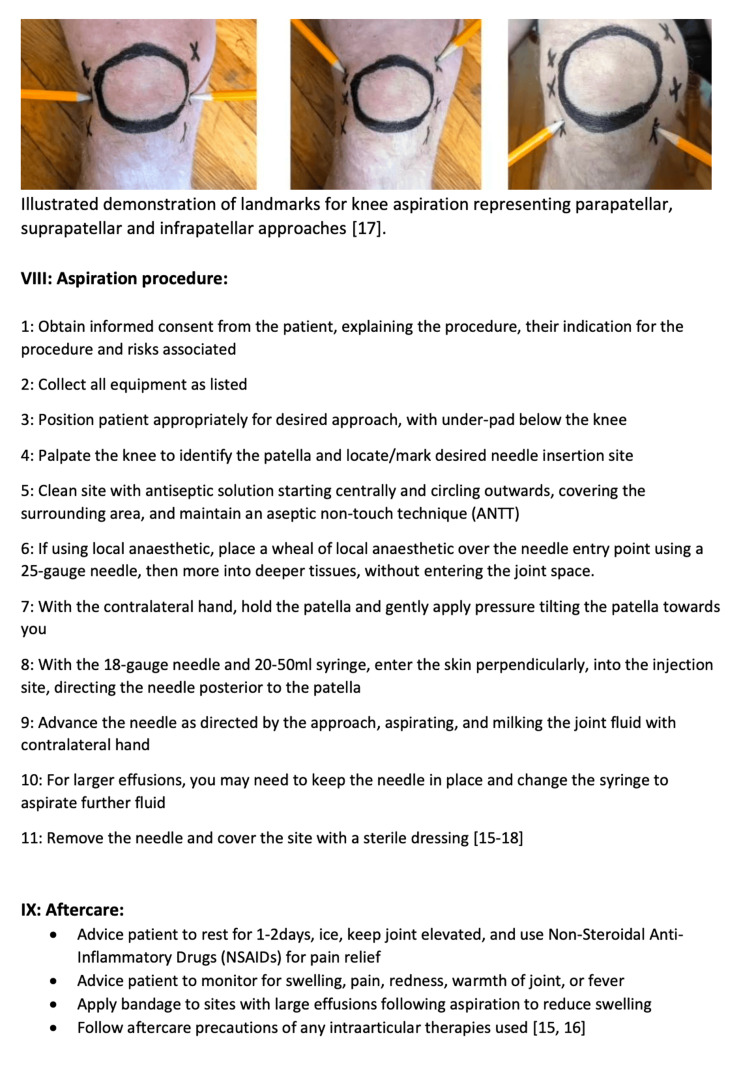
A guide to knee arthrocentesis (3/4)

**Figure 8:**
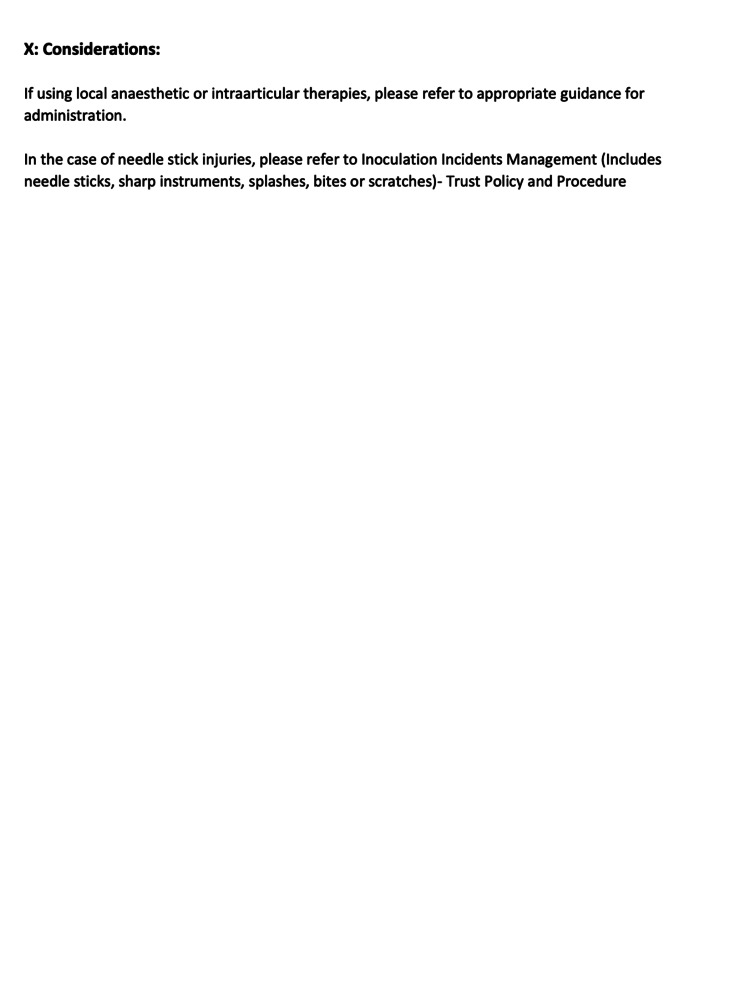
A guide to knee arthrocentesis (4/4)

**Figure 9:**
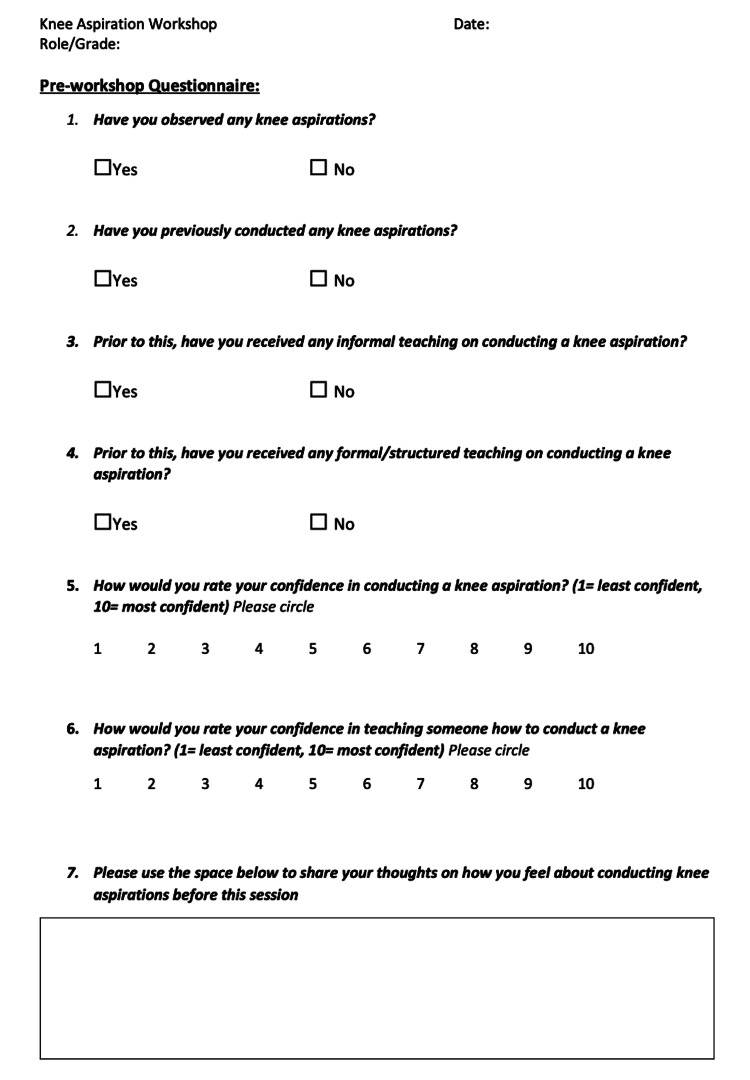
Pre-workshop questionnaire

**Figure 10:**
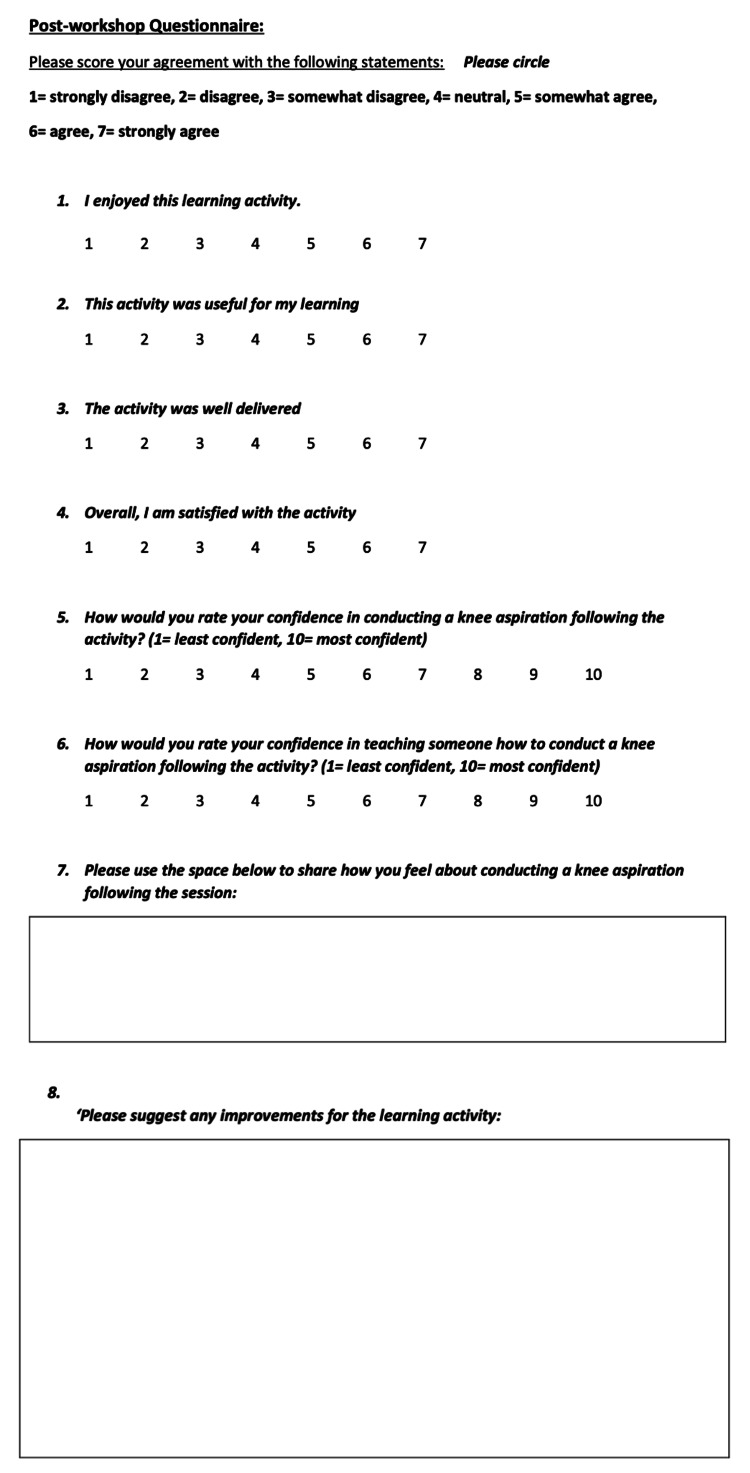
Post-workshop questionnaire

## Appendices

Appendix A

**Table 2:** Preliminary questionnaire ED: Emergency department

During your four-month block, did you observe any joint aspirations?	During your four-month block, did you conduct any joint aspirations yourself?	During your four-month block, did you have any formal teaching on the process of conducting a Joint Aspiration, i.e., lecture/seminar?	If yes to above, please provide further information on the type of teaching.	During your four-month block, did you have any informal teaching on the process of conducting a Joint Aspiration i.e. bedside teaching, 'see one, do one, teach one'?	If yes to above, please provide further information on the type of teaching.	If you answered no to having formal or informal teaching, would you have liked to have teaching (formal or informal) on conducting a Joint Aspiration?	On a scale of 1-10 (1 least confident, 10 most confident), how would you rate your confidence in conducting a Joint Aspiration?	On a scale of 1-10 (1 least confident, 10 most confident), how would you rate your confidence in teaching someone how to conduct a Joint Aspiration?
Yes	No	No	N/A	Yes	Observed 1 in ED but didn't get to do 1	Yes	1	1
No	No	No	Nil	No	Nil	Yes	2	2
Yes	No	No	-	Yes	Registrars explained procedure as they performed it	N/A	5	3
Yes	No	No	(Shouldn’t be mandatory because I put no)	No	(Shouldn’t be mandatory because I put no)	Yes	2	1
Yes	No	No	-	No	-	Yes	1	1
Yes	Yes	No	n/a	Yes	Bedside teaching during patient example	Yes	4	2

Appendix B

Appendix C 

Appendix D 

Appendix E

**Table 3:** Table of results JCF: Junior Clinical Fellow doctors; FY2: Foundation Year 2 doctors; CT1: Core Trainee 1 doctor; SHO: Senior house officer

Grade	Have you observed any knee aspirations?	Have you conducted any knee aspirations?	Prior to this, have you received any informal teaching on conducting a knee aspiration?	Prior to this, have you received any formal/structured teaching on conducting a knee aspiration?	How would you rate your confidence in conducting a knee aspiration? (1 = least confident, 10 = most confident)	How would you rate your confidence in teaching someone how to conduct a knee aspiration? (1= least confident, 10= most confident)	Please use the space below to share your thoughts on how you feel about conducting knee aspirations before this session.	I enjoyed this learning activity.	This activity was useful for my learning.	The activity was well delivered.	Overall, I am satisfied with the activity.	How would you rate your confidence in conducting a knee aspiration following the activity? (1 = least confident, 10 = most confident)	How would you rate your confidence in teaching someone how to conduct a knee aspiration following the activity? (1 = least confident, 10 = most confident)	Please use the space below to share how you feel about conducting a knee aspiration following the session.	Please suggest any improvements for the learning activity.
JCF	Yes	Yes	Yes	No	8	8	.	7	7	6	7	9	9	.	.
JCF	Yes	Yes	Yes	No	8	7	Comfortable	7	7	7	7	8	8	Useful and concise teaching	.
FY2	Yes	Yes	No	No	3	1	I have previously done a knee aspiration, however would not feel confident enough to do this independently	7	7	7	7	9	8	I feel following the session more confident in aspirating a knee joint	Better model
FY2	No	No	No	No	1	1	Scared, nervous	7	6	7	7	8	6	Thank you. I feel more capable and that I would be able to perform this procedure.	.
FY2	Yes	No	Yes	No	4	2	Nervous	7	7	7	7	7	7	More confident	.
FY2	Yes	Yes	No	No	3	1	Not very confident	7	7	7	7	8	8	Feels straight forward. Need to practice on a patient now to consolidate learning	Add to SHO handover
FY2	No	No	No	No	2	1	I haven't seen one, so would feel uncomfortable performing one	7	7	7	7	6	5	Much more confident. I would want to see one in person before I feel more confident	Really helpful, thank you
CT1	Yes	Yes	No	No	2	2	.	7	7	7	7	10	8	Increased confidence in the procedure	Found the model difficult to use

## References

[1] Abraham S, Patel S (2023). Monoarticular arthritis. StatPearls [Internet].

[2] Coakley G, Mathews C, Field M (2006). BSR & BHPR, BOA, RCGP and BSAC guidelines for management of the hot swollen joint in adults. Rheumatol.

[3] Rutherford AI, Subesinghe S, Bharucha T, Ibrahim F, Kleymann A, Galloway JB (2016). A population study of the reported incidence of native joint septic arthritis in the United Kingdom between 1998 and 2013. Rheumatology (Oxford).

[4] He Q, Mok TN, Sin TH (2023). Global, regional, and national prevalence of gout from 1990 to 2019: age-period-cohort analysis with future burden prediction. JMIR Public Health Surveill.

[5] Abram SG, Alvand A, Judge A, Beard DJ, Price AJ (2020). Mortality and adverse joint outcomes following septic arthritis of the native knee: a longitudinal cohort study of patients receiving arthroscopic washout. Lancet Infect Dis.

[6] (2024). Scenario: management of knee pain. https://cks.nice.org.uk/topics/knee-pain-assessment/management/management/.

[7] Farah Z, Reddy V, Matthews W, Giles I (2015). Poor adherence to guidelines on early management of acute hot swollen joint(s): an evaluation of clinical practice and implications for training. Int J Clin Pract.

[8] Farah Z (2017). The views and perceptions of non-specialist, hospital junior doctors on joint aspiration of the acute hot-swollen-joint, and their training in this clinical skill. Ann Rheum Dis.

[9] Kolb DA (1984). Experiential Learning: Experience as the Source of Learning and Development. https://www.researchgate.net/publication/235701029_Experiential_Learning_Experience_As_The_Source_Of_Learning_And_Development.

[10] Sawyer T, White M, Zaveri P (2015). Learn, see, practice, prove, do, maintain: an evidence-based pedagogical framework for procedural skill training in medicine. Acad Med.

[11] Burgess A, van Diggele C, Roberts C, Mellis C (2020). Tips for teaching procedural skills. BMC Med Educ.

[12] Shabbir SS, Abayalingam M, Carby M (2015). Aspirating a knee joint: a simple approach to acquiring core medical competencies. Educ Med J.

[13] Kouranloo K, Christie J (2024). Acute hot joints on the medical take: tapping into the skills of our workforce. Rheumatol Adv Pract.

[14] (2024). Knee aspiration & injection trainer with ultrasound capabilities. https://limbsandthings.com/uk/products/70103/70103-knee-aspiration-injection-trainer-with-ultrasound-capabilities.

[15] Akbarnia H, Saber AY, Smith T, Zahn E (2024). StatPearls [Internet]. https://www.ncbi.nlm.nih.gov/books/NBK470229/.

[16] (2023). How to do knee arthrocentesis. https://www.msdmanuals.com/en-gb/professional/musculoskeletal-and-connective-tissue-disorders/how-to-do-arthrocentesis/how-to-do-knee-arthrocentesis.

[17] (2021). Basics of knee aspiration. https://brownemblog.com/blogposts/2021/4/8/basics-of-knee-aspiration.

[18] Zuber TJ (2002). Knee joint aspiration and injection. Am Fam Physician.

[19] Kirkpatrick D, Kirkpatrick J (2006). Evaluating Training Programs: The Four Levels. https://scholar.google.com/scholar?hl=en&as_sdt=0%2C5&q=Kirkpatrick+DL%3A+Evaluating+Training+Programs%3A+The+Four+Levels+1996&btnG=.

